# Supplementary material for: A Two-Step Mechanism for Creating Stable, Condensed Chromatin with the Polycomb Complex PRC1
Source: Molecules. 2024 Jan 9;29(2):323. doi: 10.3390/molecules29020323 (PMC10821450; doi:10.3390/molecules29020323)
Supplement: Supplementary file 1 [file molecules-29-00323-s001.zip › supplementary/sup_figures_final.pdf]

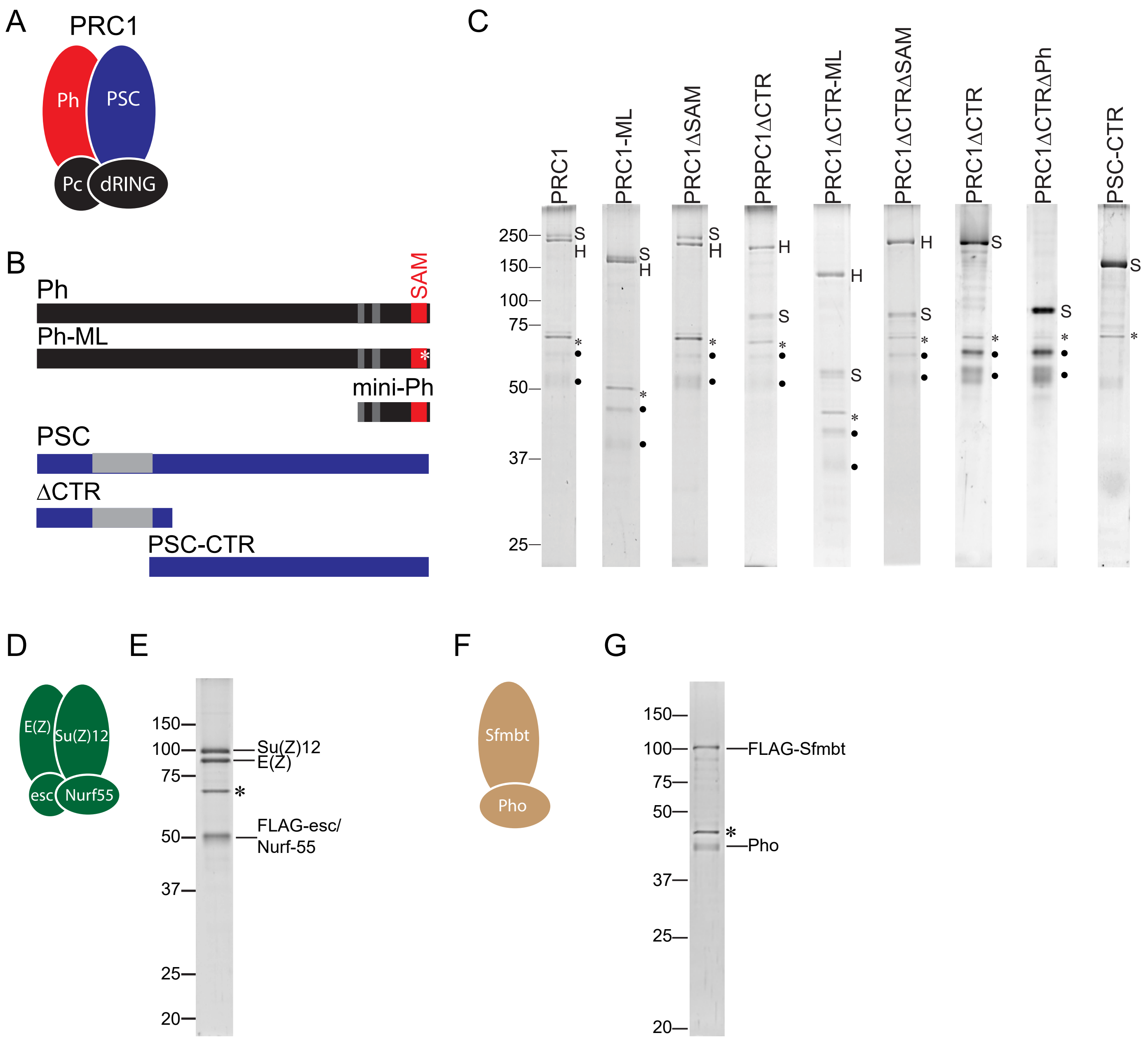
A
PRC1
Ph
PSC
Pc
dRING
B
Ph
SAM
Ph-ML
*
mini-Ph
PSC
ΔCTR
PSC-CTR
C
PRC1
PRC1-ML
PRC1ΔSAM
PRPC1ΔCTR
PRC1ΔCTR-ML
PRC1ΔCTRΔSAM
PRC1ΔCTR
PRC1ΔCTRΔPh
PSC-CTR
250
150
100
75
50
37
25
S
H
*
D
E(Z)
Su(Z)12
esc
Nurf55
E
150
100
75
50
37
25
20
Su(Z)12
E(Z)
*
FLAG-esc/
Nurf-55
F
Sfmbt
Pho
G
150
100
75
50
37
25
20
FLAG-Sfmbt
*
Pho

Figure S1 Protein preparations. A. Schematic of PRC1. B. Domain organization of Ph and PSC and truncations/mutations used. Gray regions and the SAM are structured domains; the rest of both proteins is predicted or shown to be disordered. C. SDS-PAGE gel stained with SYPRO Ruby showing different PRC1 variants of Ph and/or lacking the PSC-CTR, and the PSC-CTR alone. S indicates the position of PSC, H the position of Ph. Asterisk is co-purifying Hsc70 and filled circles indicate Pc (top) and dRING (bottom). Lanes are from different gels; marker is only shown for the first lane. D, E. Schematic (D) and SYPRO Ruby-stained SDS-PAGE (E) of PRC2. F, G. Schematic(F) and SYPRO Ruby stained SDS-PAGE (G) of PhoRC. Asterisk in E and F indicates co-purifying Hsc70.

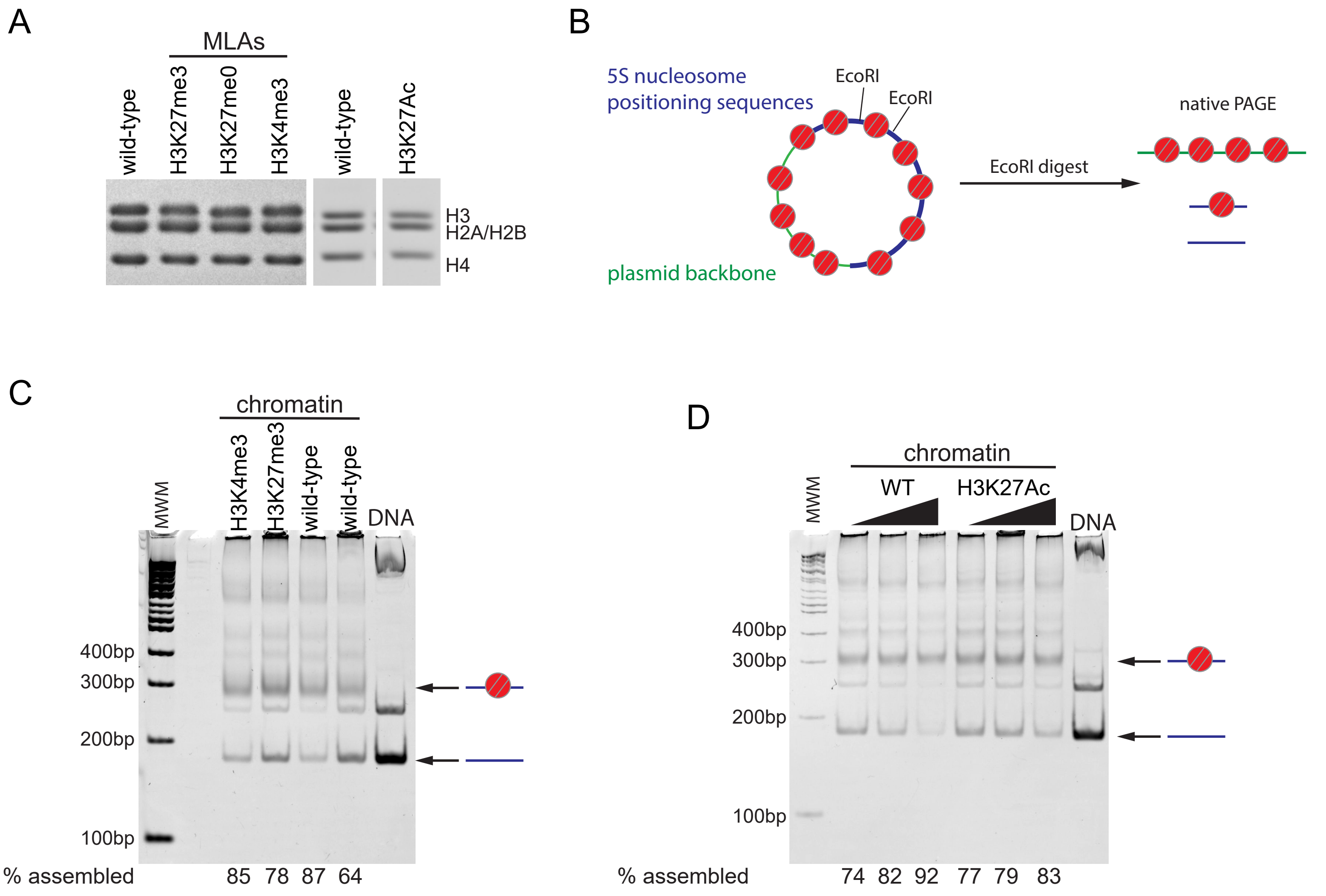

A
MLAs
wild-type
H3K27me3
H3K27me0
H3K4me3
wild-type
H3K27Ac
H3
H2A/H2B
H4
B
5S nucleosome
positioning sequences
EcoRI
EcoRI
plasmid backbone
EcoRI digest
native PAGE
C
chromatin
MWM
H3K4me3
H3K27me3
wild-type
wild-type
DNA
400bp
300bp
200bp
100bp
% assembled
85 78 87 64
D
chromatin
WT
H3K27Ac
MWM
DNA
400bp
300bp
200bp
100bp
% assembled
74 82 92 77 79 83

Figure S2 Chromatin preparation. A. Coomassie blue stained SDS-PAGE of reconstituted histone octamers with the indicated modifications. B. Schematic of the EcoRI digest assay to measure nucleosome assembly over the 5S repeats (taken from [35]). C, D. EcoRI analysis of wild type and MLA-containing chromatin (C), and wild type and H3K27Ac chromatin (D).

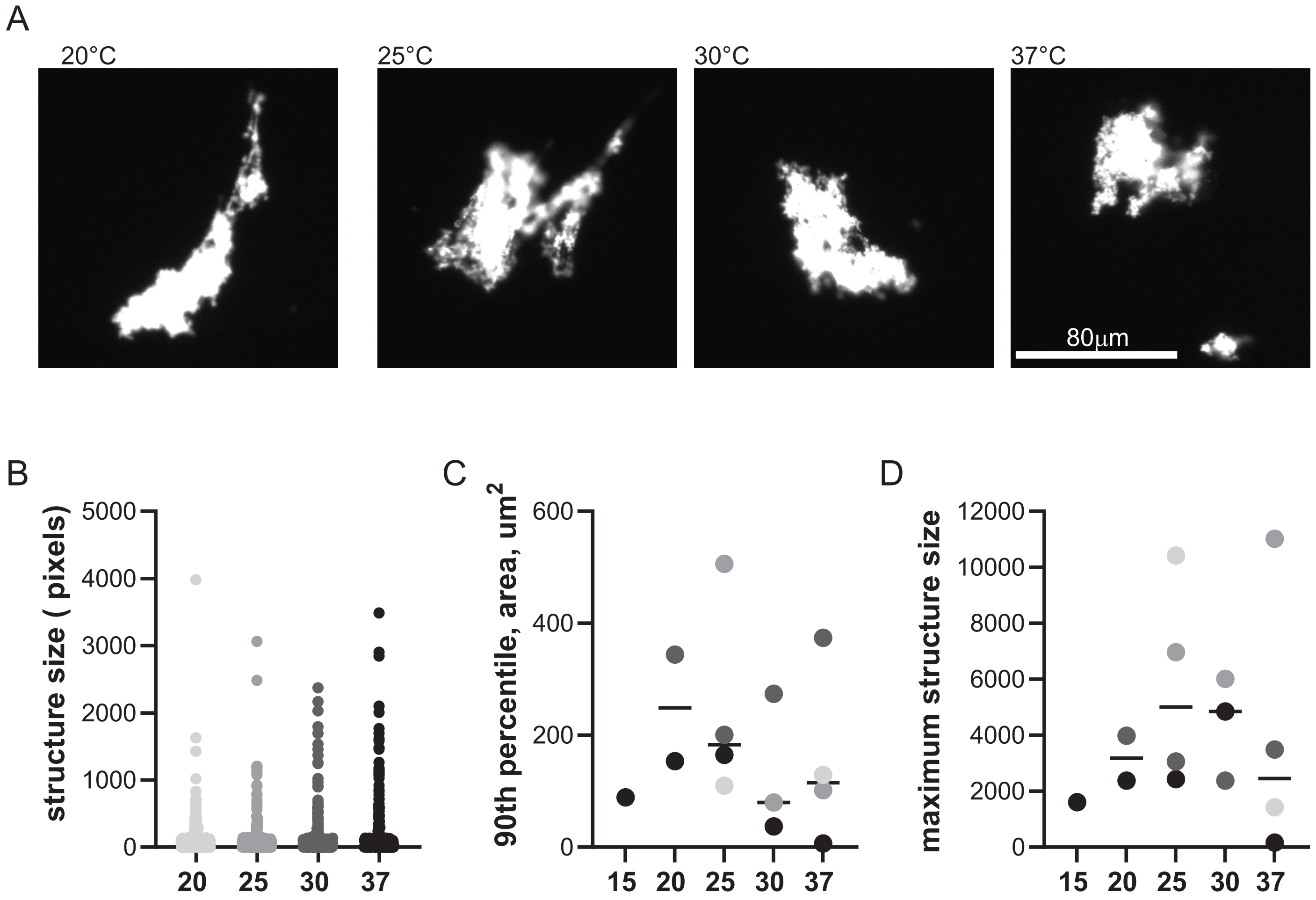
A
20°C
25°C
30°C
37°C
80μm
B
structure size (pixels)
5000
4000
3000
2000
1000
0
20
25
30
37
C
90th percentile, area, um2
600
400
200
0
15
20
25
30
37
D
maximum structure size
12000
10000
8000
6000
4000
2000
0
15
20
25
30
37

Figure S3 Effect of temperature on PRC1-chromatin condensate formation. A. Images of structures formed by PRC1 + chromatin during overnight incubation at indicated temperatures. B. Quantification of structures formed at different temperatures for a representative experiment. No differences between pairs of samples (i.e. 20-25, 25-30, 30-37) were detected by Kruskal-Wallace test with Dunn's correction for multiple comparisons. C, D. Summary of structures formed at different temperatures, showing the 90th percentile (C) and maximum (D) area of structures formed. Symbols that are the same shade are from the same experiment.

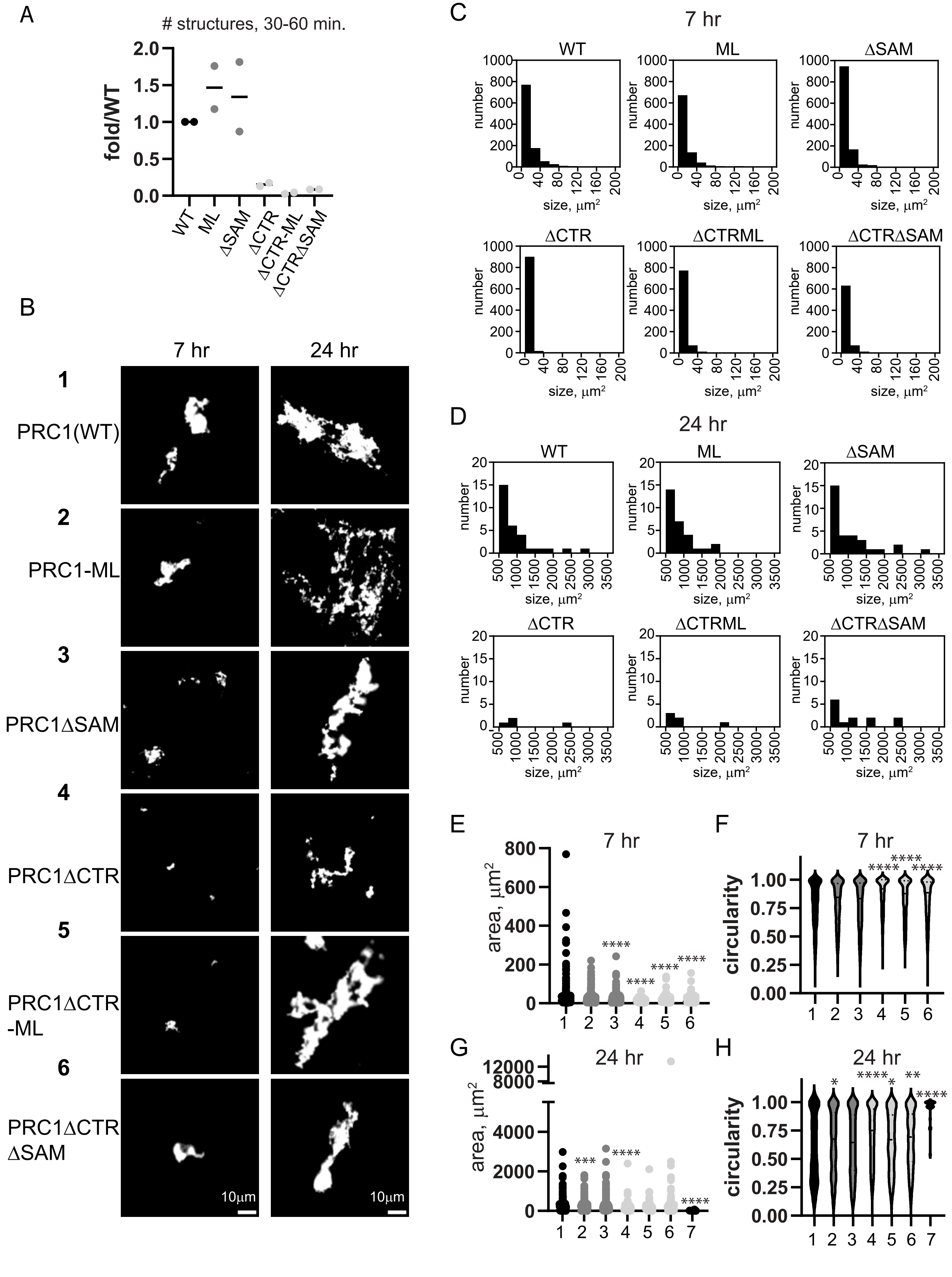
A
structures, 30-60 min.
fold/WT
2.0
1.5
1.0
0.5
0.0
WT
ML
ΔSAM
ΔCTR
ΔCTR-ML
ΔCTRΔSAM
B
7 hr
24 hr
1
PRC1(WT)
2
PRC1-ML
3
PRC1ΔSAM
4
PRC1ΔCTR
5
PRC1ΔCTR
-ML
6
PRC1ΔCTR
ΔSAM
10μm
10μm
C
7 hr
WT
ML
ΔSAM
ΔCTR
ΔCTRML
ΔCTRΔSAM
number
size, μm²
D
24 hr
WT
ML
ΔSAM
ΔCTR
ΔCTRML
ΔCTRΔSAM
number
size, μm²
E
7 hr
area, μm²
1 2 3 4 5 6
F
7 hr
circularity
1 2 3 4 5 6
G
24 hr
area, μm²
1 2 3 4 5 6 7
H
24 hr
circularity
1 2 3 4 5 6 7

Figure S4 The PSC-CTR increases the rate of formation and size of PRC1-chromatin condensates. A. Quantification of structures formed at short time points (30 or 60 min.) by different complexes in two different experiments. Numbers were normalized to the number in reactions with wild-type PRC1. B. Representative structures formed with different complexes after 7 or 24 hours. C. Histograms of the number structures formed by different complexes after 7 hours of incubation. D. Histograms showing the number of structures greater than 500μm^2 formed by different complexes with chromatin after 24 hours. Chromatin graph is not shown as no structures in this size range were observed. E-H. Quantification of area (E, G) or circularity (F, H) of structures formed by different complexes at different time points. Asterisks are for Kruskal-Wallace test with Dunn's correction for multiple comparisons (****=$p \leq 0.0001$). See **Table S1** for a summary of comparisons of area and circularity across multiple experiments.

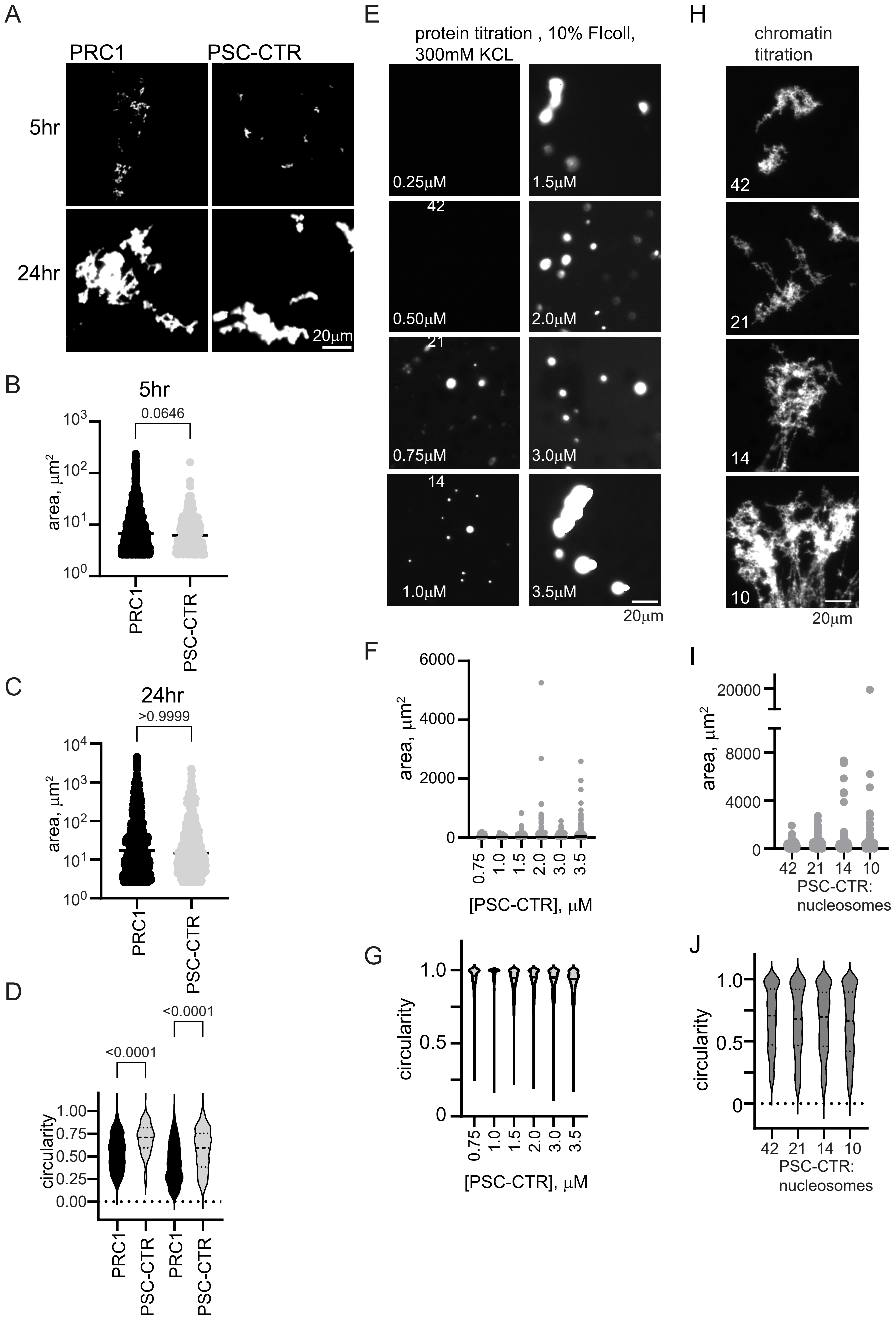

A
PRC1
PSC-CTR
5hr
24hr
20µm
B
5hr
0.0646
area, µm²
PRC1
PSC-CTR
C
24hr
>0.9999
area, µm²
PRC1
PSC-CTR
D
<0.0001
<0.0001
circularity
PRC1
PSC-CTR
PRC1
PSC-CTR
E
protein titration , 10% FIcoll, 300mM KCL
0.25µM
1.5µM
42
0.50µM
2.0µM
21
0.75µM
3.0µM
14
1.0µM
3.5µM
20µm
F
area, µm²
[PSC-CTR], µM
G
circularity
[PSC-CTR], µM
H
chromatin titration
42
21
14
10
20µm
I
area, µm²
PSC-CTR: nucleosomes
J
circularity
PSC-CTR: nucleosomes

Figure S5 The PSC-CTR forms round condensates with Ficoll, and large networks with chromatin at 120mM KCl. A. Representative images of structures formed by PRC1 (10nM) or the PSC-CTR (80nM) with chromatin (replicate of experiment shown in **Figure 4A-D**). B-D. Quantification of structure areas after 5 (B) or 22 (C) hours, and of circularity (D) for both time points. E. Condensates formed by the PSC-CTR in the presence of Ficoll (100mg/ml). F, G. Quantification of area (F) and circularity (G) of titration shown in E. H. Titration of chromatin with the PSC-CTR (0.72µM). Number indicates the ratio of protein to nucleosomes, which were used at ~17, 34, 52, and 69nM. I, J. Quantification of area (I) and circularity (J) of titration shown in H, indicating that structures increase in size with increased addition of chromatin.

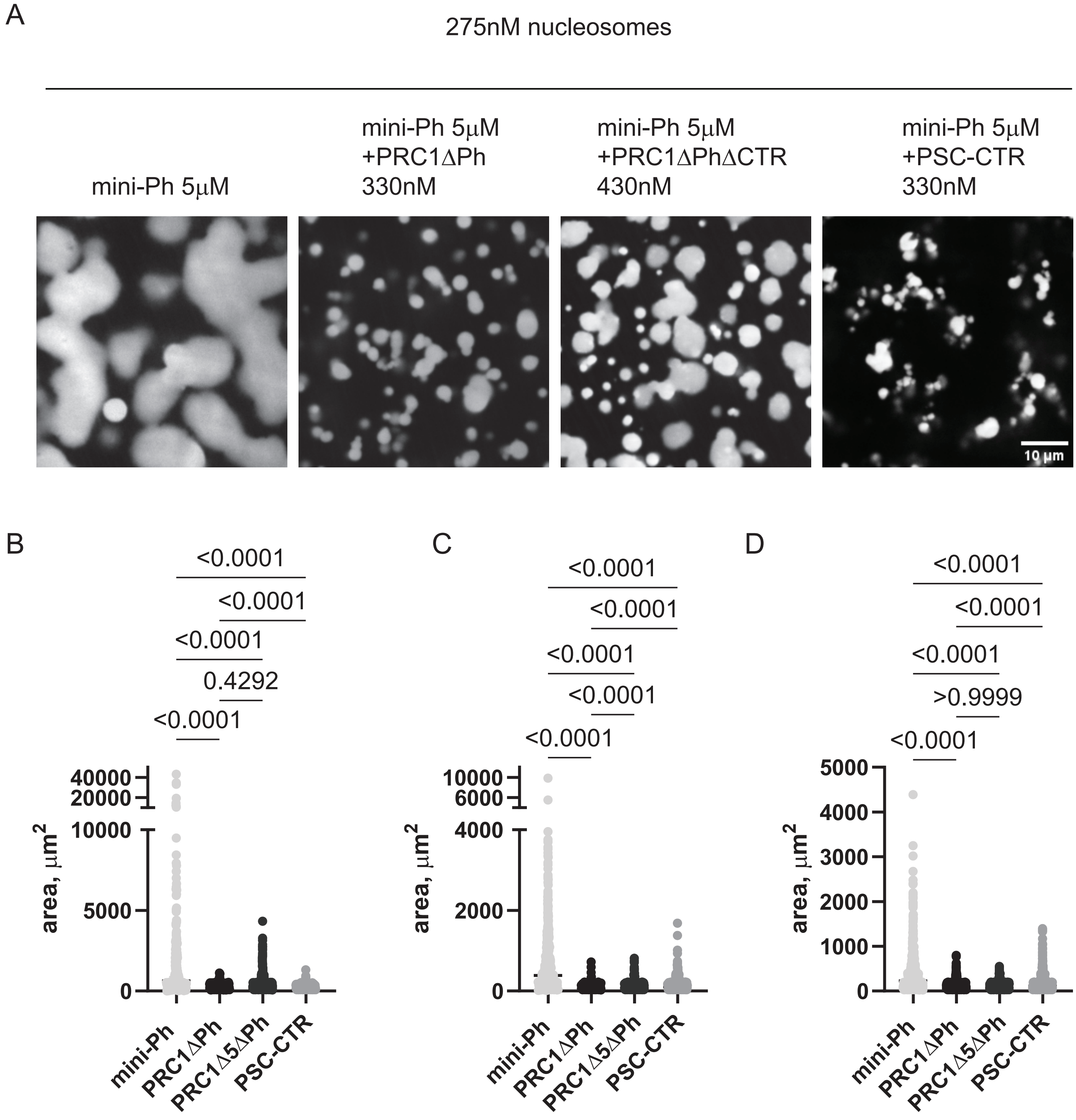
A
275nM nucleosomes
mini-Ph 5μM
mini-Ph 5μM
+PRC1ΔPh
330nM
mini-Ph 5μM
+PRC1ΔPhΔCTR
430nM
mini-Ph 5μM
+PSC-CTR
330nM
10 μm
B
<0.0001
<0.0001
<0.0001
0.4292
<0.0001
40000
20000
10000
5000
0
area, μm²
mini-Ph
PRC1ΔPh
PRC1Δ5ΔPh
PSC-CTR
C
<0.0001
<0.0001
<0.0001
<0.0001
<0.0001
10000
6000
4000
2000
0
area, μm²
mini-Ph
PRC1ΔPh
PRC1Δ5ΔPh
PSC-CTR
D
<0.0001
<0.0001
<0.0001
>0.9999
<0.0001
5000
4000
3000
2000
1000
0
area, μm²
mini-Ph
PRC1ΔPh
PRC1Δ5ΔPh
PSC-CTR

Figure S6 PRC1 lacking the PSC-CTR requires higher concentrations to arrest mini-Ph-chromatin condensates. A. Representative images of reactions with 5mM mini-Ph and 275nM nucleosomes after addition of buffer, PRC1ΔPh, PRC1ΔCTRΔPH, or the PSC-CTR at the indicated concentrations. B-D. Quantification of the area of condensates in three replicates of the experiment shown in A. p-values are for Kruskal-Wallace test with Dunn's correction for multiple comparisons.

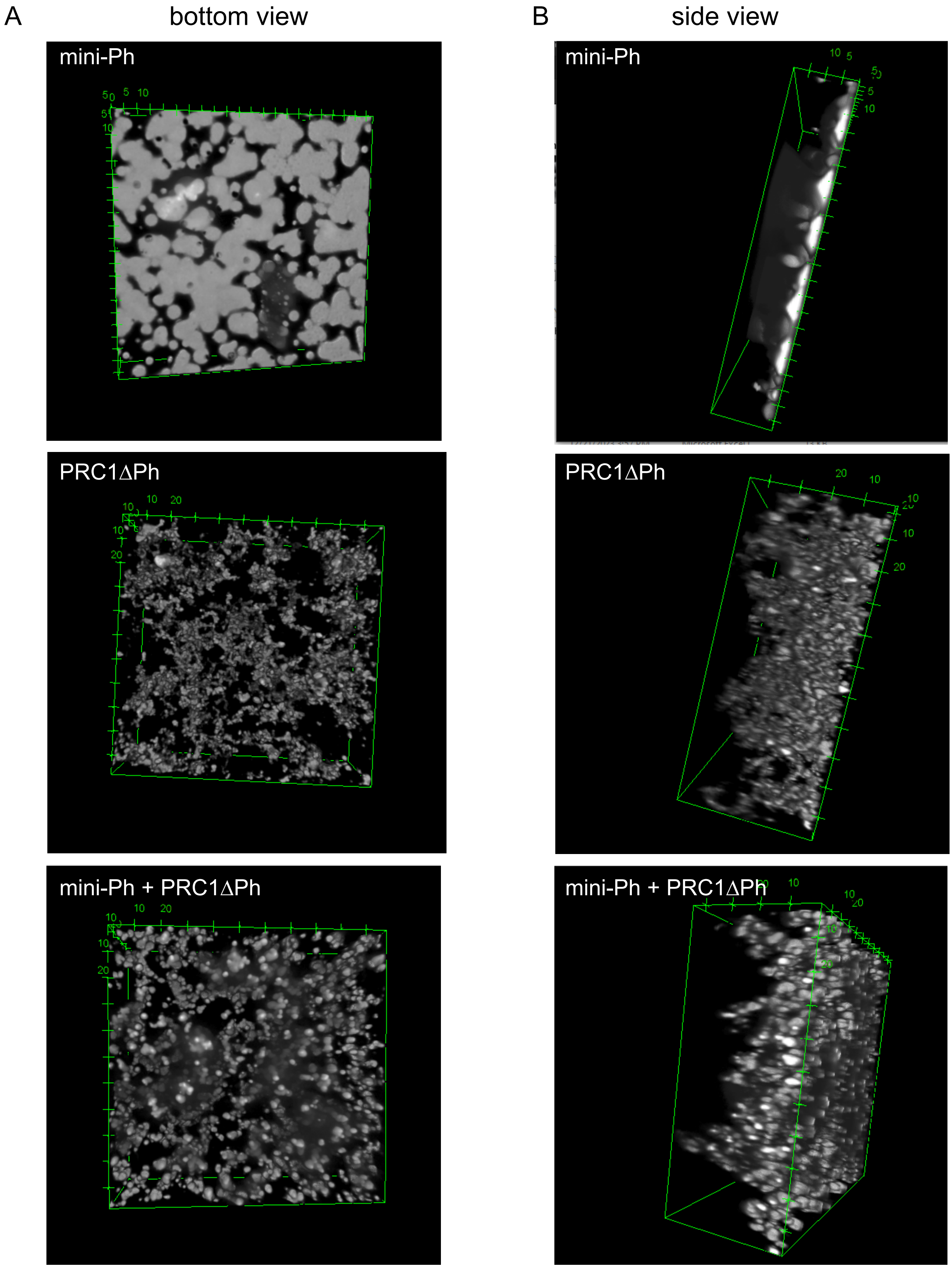
A
bottom view
B
side view
mini-Ph
PRC1ΔPh
mini-Ph + PRC1ΔPh
mini-Ph
PRC1ΔPh
mini-Ph + PRC1ΔPh

Figure S7 3D volume views of structures shown in Figure 6. A. 3D-volume view from the bottom of the confocal stack. B. Side view of confocal stack indicating that mini-Ph chromatin condensates are flattened onto the glass surface while fibers formed with PRC1DPh extend into solution along the z-axis.

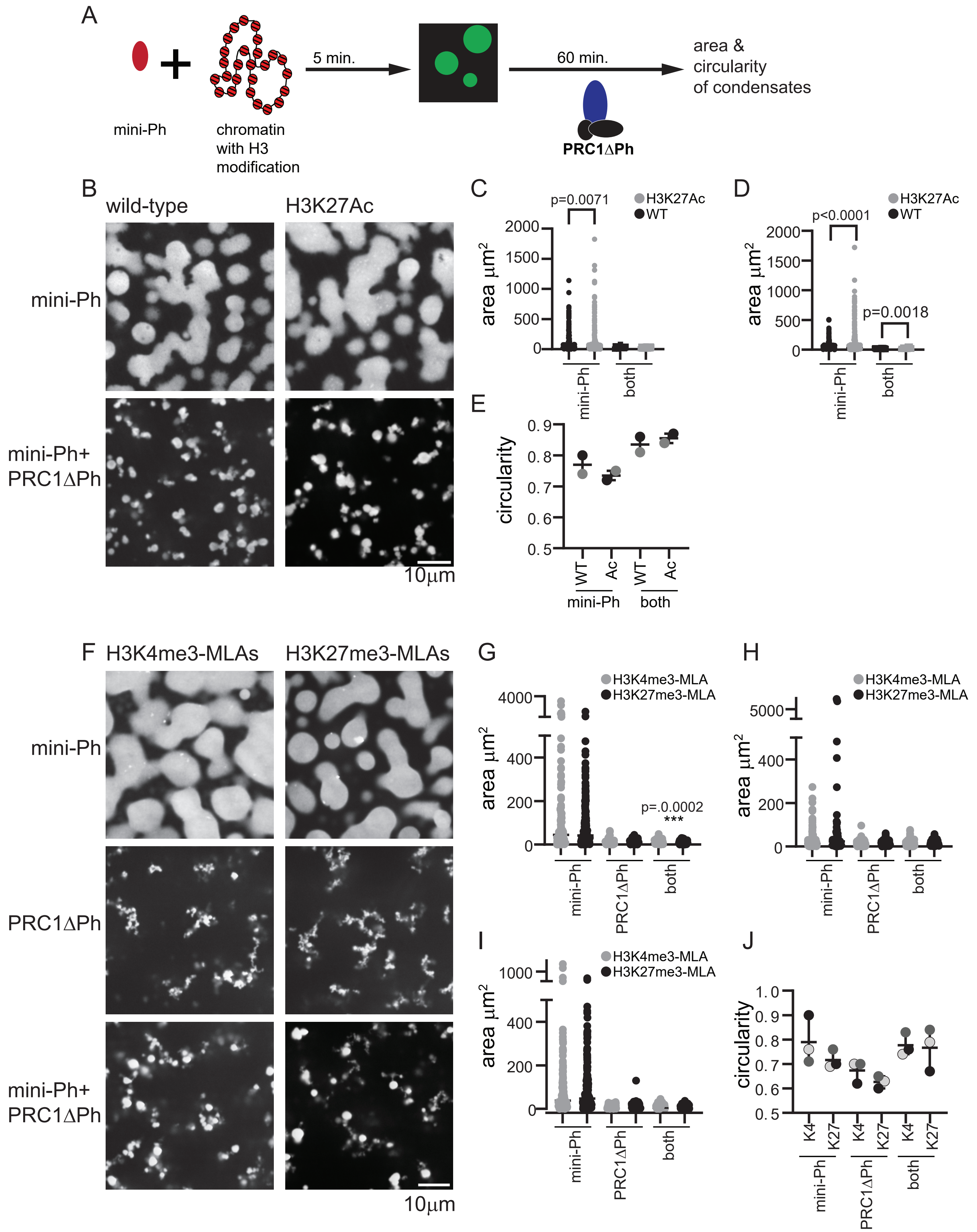
A
5 min.
60 min.
area &
circularity
of condensates
mini-Ph
chromatin
with H3
modification
PRC1ΔPh
B
wild-type
H3K27Ac
mini-Ph
mini-Ph+
PRC1ΔPh
10μm
C
p=0.0071
H3K27Ac
WT
area μm2
mini-Ph
both
D
p<0.0001
p=0.0018
E
circularity
WT
Ac
F
H3K4me3-MLAs
H3K27me3-MLAs
PRC1ΔPh
G
H3K4me3-MLA
H3K27me3-MLA
p=.0.0002
***
H
I
J
K4
K27

Figure S8. Methylation of H3K4 or K3K27, or acetylation of H3K27 do not impair or enhance PRC1ΔPh or mini-Ph activity. A. Schematic of two step protocol. B. Representative images of condensates formed with mini-Ph or mini-Ph + PRC1ΔPh with wild type or H3K27Ac chromatin. C, D. Quantification of condensate areas for independent experiments. E. Median circularity for each reaction from two experiments. Medians from the same experiment are shaded the same. F. Representative images of condensates formed with mini-Ph or mini-Ph + PRC1ΔPh with chromatin containing H3K4me3 or H3K27me3 methyl lysine analogues (MLA). G-I. Condensate areas for replicate experiments. Asterisks and p-values are for Kruskal-Wallace test with Dunn's correction for multiple comparisons. In each case, comparisons were done between H3K4me3 and H3K27me3 chromatin incubated with the same proteins. J. Plot of the median circularity from three replicate experiments. Values from the same experiment are shaded the same. For all experiments, mini-Ph was used at 4µM, PRC1ΔPh at 0.4µM, and nucleosomes at ~0.33uM. Condensates were visualized by Cy3 on H2A. Chromatin assemblies were: WT=82%; K27Ac=83%; H34me3=85%; H3K27me3=78%.

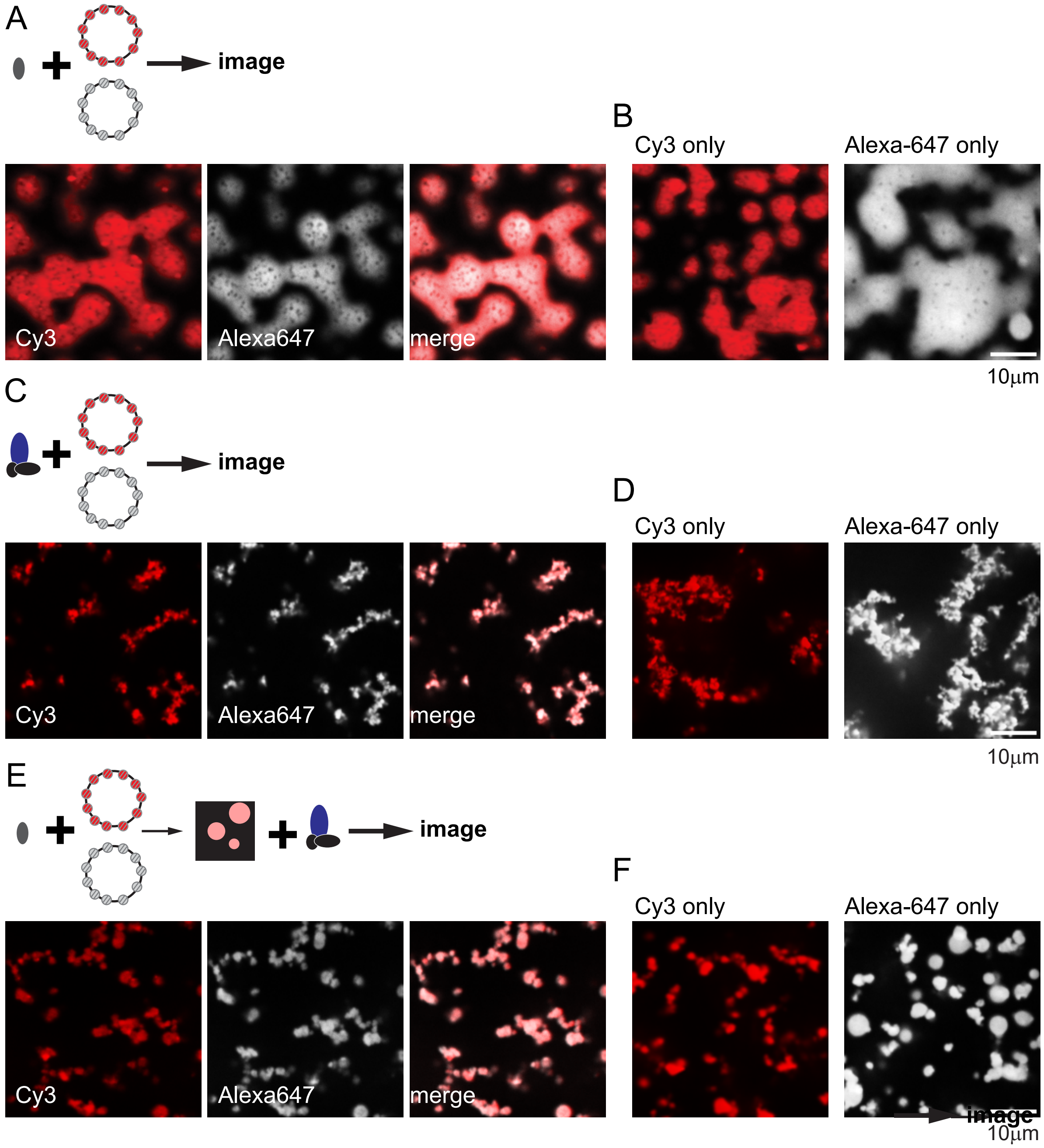
A
image
B
Cy3 only
Alexa-647 only
Cy3
Alexa647
merge
10µm
C
image
D
Cy3 only
Alexa-647 only
Cy3
Alexa647
merge
10µm
E
image
F
Cy3 only
Alexa-647 only
Cy3
Alexa647
merge
image
10µm

Figure S9 Control experiments for mixing experiments with different chromatin templates. A, C, E show representative images of reactions where both templates are added prior to adding the indicated protein. B, D, F show images of reactions with single template.

A

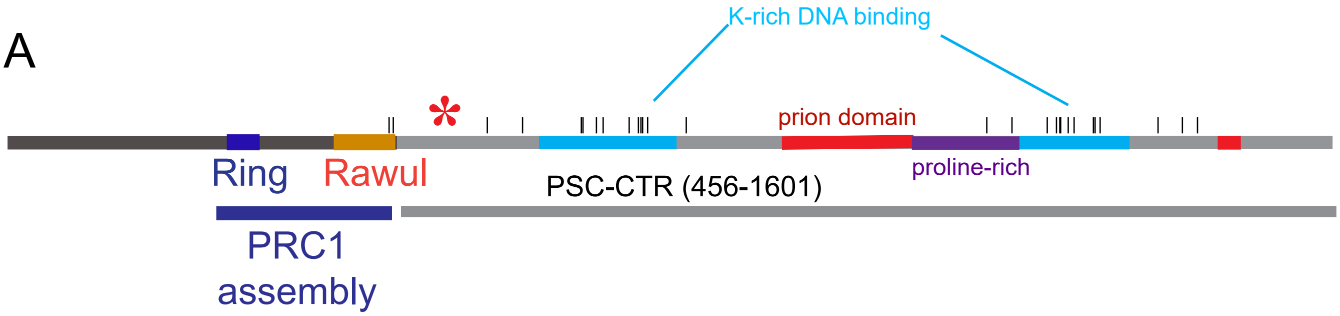

B sequence of prion domain:

small non-polar
small polar
aromatic
positively charged
negatively charged
proline

PNSPIYSPSS PQYVPSYNIP TMPTYKYTPK PTPNSGSGNG GSGSYLQNML
GGGNGGSLGG LFPSPPTKSD QNTNPAQGGG GSSSATQSGG NNGIVNNNIY
MPN

Figure S10 Additional analysis of the PSC-CTR. A. Sequence and functional features in the PSC-CTR. The two putative DNA binding regions were identified in protein footprinting experiments [46], while proline rich and prion domains were identified by sequence analysis. PLAAC [57] was used to identify the prion domains. The region between the two DNA binding domains forms many contacts with other parts of PSC in cross-linking mass spec experiments [46]. Black lines indicate phosphorylation sites identified in our AP-MS analysis (although not independently validated) or available on UniProt. Asterisk indicates position of a truncation mutation that is lethal in *Drosophila* embryos [18, 42]. B. Sequence of the main prion domain.
